# Important contributions of sea-salt aerosols to atmospheric bromine cycle in the Antarctic coasts

**DOI:** 10.1038/s41598-018-32287-4

**Published:** 2018-09-14

**Authors:** Keiichiro Hara, Kazuo Osada, Masanori Yabuki, Hisahiro Takashima, Nicolas Theys, Takashi Yamanouchi

**Affiliations:** 1 Department of Earth System Science, Faculty of Science, Fukuoka University, Fukuoka, Japan; 2 Graduate School of Environmental Studies, Nagoya University, Nagoya, Japan; 3 Research Institute for Sustainable Humanosphere, Kyoto University, Uji, Kyoto, Japan; 4 Belgian Institute for Space Aeronomy, Brussels, Belgium; 5 National Institute of Polar Research, Tokyo, Japan

## Abstract

Polar sunrise activates reactive bromine (BrO_x_) cycle on the Antarctic coasts. BrO_x_ chemistry relates to depletion of O_3_ and Hg in polar regions. Earlier studies have indicated “blowing snow” as a source of atmospheric BrO_x_. However, surface O_3_ depletion and BrO enhancement occurs rarely under blowing snow conditions at Syowa Station, Antarctica. Therefore, trigger processes for BrO_x_ activation other than the heterogeneous reactions on blowing snow particles must be considered. Results of this study show that enhancement of sea-salt aerosols (SSA) and heterogeneous reactions on SSA are the main key processes for atmospheric BrO_x_ cycle activation. Blowing snow had Br^−^ enrichment, in contrast to strong Br^−^ depletion in SSA. *In-situ* aerosol measurements and satellite BrO measurements demonstrated clearly that a BrO plume appeared simultaneously in SSA enhancement near the surface. Results show that surface O_3_ depletion at Syowa Station occurred in aerosol enhancement because of SSA dispersion during the polar sunrise. Amounts of depleted Br^−^ from SSA were matched well to the tropospheric vertical column density of BrO and BrO_x_ concentrations found in earlier work. Our results indicate that SSA enhancement by strong winds engenders activation of atmospheric BrO_x_ cycles via heterogeneous reactions on SSA.

## Introduction

The reactive bromine (BrO_x_) cycle is activated in polar regions during the polar sunrise^1–3^. The atmospheric BrO_x_ cycle relates to atmospheric chemistry such as depletion of O_3_ (R1) and Hg, and oxidation of dimethylsulfide^1,2,4,5^.

R1 $${\rm{Br}}+{{\rm{O}}}_{3}\to {\rm{BrO}}+{{\rm{O}}}_{2}$$

Results of earlier studies have shown that BrO_x_ origins are heterogeneous reactions (R2–3) occurring on the surfaces of blowing snow, sea-salt aerosols (SSA), frost flowers, and surface snow^6–9^ and subsequent photolysis of Br_2_ and BrCl (R4–5).

R2 $${\rm{HOBr}}+{{\rm{Br}}}^{-}+{{\rm{H}}}^{+}\to {{\rm{Br}}}_{{\rm{2}}}+{{\rm{H}}}_{{\rm{2}}}{\rm{O}}$$

R3 $${\rm{HOBr}}+{{\rm{Cl}}}^{-}+{{\rm{H}}}^{+}\to {\rm{BrCl}}+{{\rm{H}}}_{{\rm{2}}}{\rm{O}}$$

R4 $${\rm{BrCl}}+{{\rm{Br}}}^{-}\to {{\rm{Br}}}_{{\rm{2}}}{{\rm{Cl}}}^{-}$$

R5 $${{\rm{Br}}}_{{\rm{2}}}{{\rm{Cl}}}^{-}\to {{\rm{Br}}}_{{\rm{2}}}+{{\rm{Cl}}}^{-}$$

Satellite measurements showed high BrO concentrations over sea-ice with the appearance of frost flowers^8^. Additionally, high BrO_x_ plumes in the Antarctic coasts were found to originate from sea-ice zones^2,10^. Model studies presented the contribution of blowing snow as a BrO_x_ source in polar regions^2,6^. The lifetime of blowing snow, however, is too short because of efficient dry deposition. Although reactions of R2 and R3 require acidity for the release of Br_2_ and BrCl as a trigger of the atmospheric BrO_x_ cycle, blowing snow has higher pH. In contrast to large amounts of acidic species supplied from anthropogenic processes in the Arctic^11^, the source strength of anthropogenic acidic species is less in the Antarctic Circle. Therefore, acidity/alkalinity in the surface snow with high salinity is likely to be different in Antarctica and the Arctic. To elucidate this discrepancy, we explore the origins of atmospheric BrO_x_ based on simultaneous measurements of aerosols and blowing snow at Syowa Station, Antarctica.

## Results and Discussion

### Short-term features of aerosol number density and BrO

**Figure 1:**
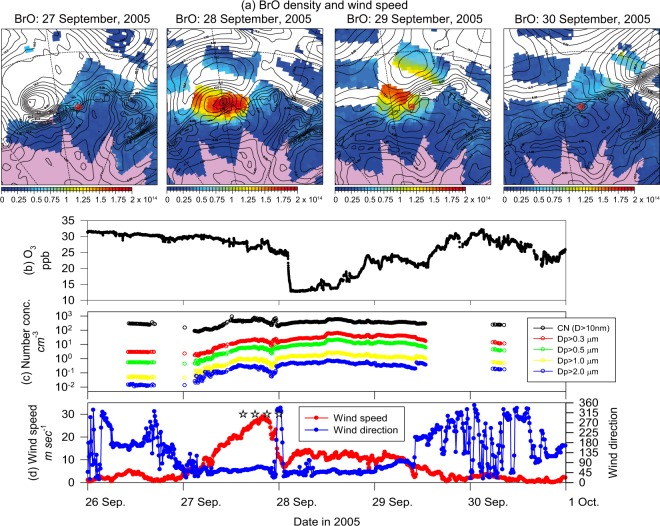
BrO mapping around Lützow-Holm Bay on 27–30 September 2005 and short-term features of the concentrations of surface O_3_, condensation nuclei, and aerosol particles larger than 0.3 μm and 1 μm, wind directions and wind speed at Syowa Station, Antarctica on 26–30 September 2005. Unit of BrO density is mole cm^−2^. Thick lines in BrO maps are wind speeds at 925 hPa in the ERA-interim. Stars in (d) show the occurrence of blowing snow observed visually every 3 hr by us and by members of the Japanese Meteorological Agency.

Blowing snow appeared on 27 September 2005 at Syowa under strong wind conditions as a cyclone approached (Fig. 1). Aerosol number concentrations increased concomitantly with the occurrence of blowing snow. Here, we designate ice particles with with *D*_p_ (diameter) > 10 μm and particles dominantly containing sea-salts with *D*_p_ < 10 μm respectively as blowing snow particles and sea-salt aerosols (SSA). Air masses around Lützow-Holm Bay passed through the boundary layer over sea-ice during the prior 5 days (Fig. S1). Previous investigations^2,6^ pointed out that blowing snow acts as a source of BrO_x_. However, satellite measurements showed that the tropospheric vertical column density of BrO (VCD_BrO_) was not markedly elevated on 27 September. Although some likelihood exists that cloud cover disturbed satellite BrO measurement near the surface, the surface O_3_ concentration dropped slightly under storm conditions on 27 September. Considering that O_3_ can be depleted by the reaction (R1) during polar sunrise, slight O_3_ depletion implies low BrO concentration near the surface. The wind speed dropped suddenly around 00UT on 28 September 2005. Blowing snow disappeared on 28 September 2005 because of the sudden decline of the cyclone, reduction of release of blowing snow particles, and the rapid deposition of blowing snow particles. Aerosol-enhanced conditions (AECs) with higher number concentrations in fine and coarse modes and without blowing snow (i.e., Antarctic haze^12^) persisted after the storm condition. Simultaneously, the O_3_ concentration dropped to 13.7 ppb. VCD_BrO_ was enhanced to the order of 10^14^ mole cm^−2^ near Syowa. The BrO-enhanced area overlapped the area with wind speed of less than 10 m s^−1^. This condition persisted until 29 September 2005. The aerosol and O_3_ concentrations recovered simultaneously to their respective background levels on 30 September, when an air mass came from the free troposphere over the continent (Fig. S1). Background aerosol number concentrations in September were 200–300 cm^−3^ in CN, 2–3 cm^−3^ in D_p_ > 0.3 μm, and 0.1–0.2 cm^−3^ in *D*_p_ > 1.0 μm^13^. This relation suggests strongly that BrO enhancement and O_3_ depletion are related to AECs rather than to appearance of blowing snow at the Antarctic coasts.

### Chemical constituents of blowing snow and aerosols on 27–29 September 2005

**Figure 2:**
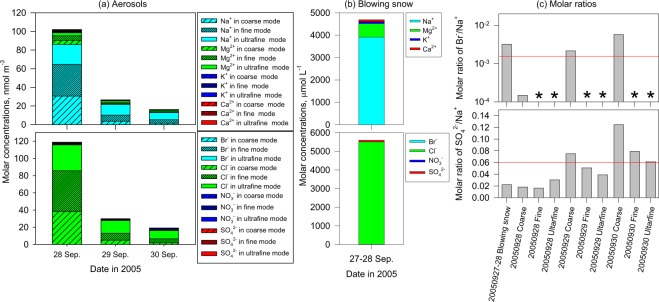
Molar concentrations of (**a**) aerosols on 28–30 September 2005, (**b**) blowing snow taken on 27–28 September 2005, and (**c**) molar ratios of SO_4_^2−^/Na^+^ in blowing snow and aerosols during 28–30 September. Coarse, fine, and ultrafine modes respectively have ranges of *Dp* > 2 μm, *Dp* = 0.2–2 μm, and *Dp* < 0.2 μm. Red lines and asterisks in (**c**), respectively represent bulk seawater ratios and data with Br^−^ concentrations below the detection limit.

Major constituents of blowing snow and aerosols in the case on 27–29 September 2005 are sea-salts (Fig. 2). Lower molar ratios of SO_4_^2−^/Na^+^ by sea-salt fractionation on sea-ice^14,15^ constitute direct evidence that sea-salts in blowing snow and aerosols originated from sea-ice. The aerosol number concentrations in the storm and AECs were much higher relative to the background levels at Syowa (Fig. 1). Therefore, SSA on 28–29 September was likely released from sea-ice area through erosion of saline snow^16^ on sea-ice by strong winds and by sublimation of snow particles^16–18^.

Data show that Br^−^ was enriched in blowing snow, whereas Br^−^/Na^+^ in aerosols was lower than seawater ratio (SWR), particularly in ultrafine – fine modes (Fig. 2). Br^−^ was depleted also from SSA in coarse mode on 28 September. Although Br^−^ release from sea-salts can be promoted in acidic conditions^1,3,19^, pH tended to increase with conductivity corresponding to sea-salt concentrations (Fig. S2). Comparison between air mass origins and the conductivity of blowing snow samples shows no clear differences (Fig. S3) because air masses in most cases flowed over sea-ice area in Lützow-Holm Bay immediately before approaching Syowa Station. When sea-ice in the Ongul Strait was broken and flowed twice in winter 2004, Syowa Station was ca. 2 km distant from seasonal sea-ice. Although multi-year sea-ice with age of 2–3 years and thickness of 56–110 cm were present in the Ongul Strait in 2005–2006, snow on the sea-ice in the strait often high salinity because of the migration of seawater through cracks during the winter. Therefore, blowing snow particles might be released from snow on sea-ice in Lützow-Holm Bay through erosion in strong winds, as suggested by earlier work^16^. Conductivity and sea-salt concentrations of blowing snow samples can be altered by mixing of snowfall particles during sampling and dilution by snowfall deposition onto the sea-ice/snow surface (before release to the atmosphere). However, it is difficult to divide pH between blowing snow particles and snowfall particles in our sampling procedures because blowing snow and snowfall occurred simultaneously in storm conditions. Because of snowfall mixing during sampling, the ambient pH of blowing snow particles is expected to be higher than the pH in blowing snow samples (Supplementary). Therefore, Br^−^ release might be reduced in blowing snow with higher pH comparing to SSA. By contrast, SSA have longer residence time and larger surface area relative to volumes. In general, the larger relative surface area in smaller particles can enhance heterogeneous reactions. Consequently, many BrO_x_ can be released from SSA through heterogeneous reactions and can be converted in the atmosphere through heterogeneous reactions (R2–5)^1,4^. Considering Br^−^ enrichment in blowing snow and strong Br^−^ depletion in SSA during AECs, we anticipate that SSA dispersion and then Br^−^ depletion in SSA through the heterogeneous reactions play important roles in the atmospheric BrO_x_ cycle in the case of 27–30 September 2005.

### Relation between AECs and surface O_3_

**Figure 3:**
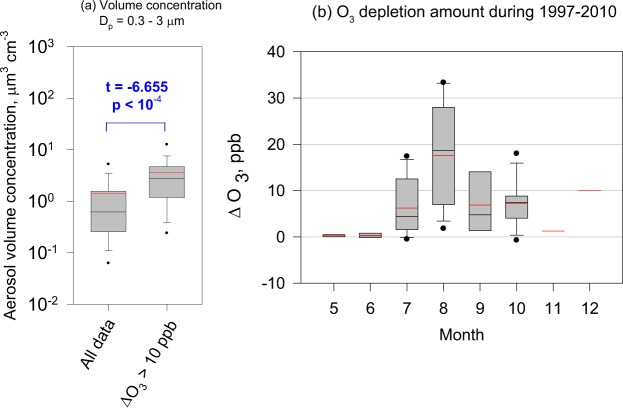
(**a**) Aerosol volume concentrations of the whole period (1997–2011) and surface ozone depletion (ΔO_3_ > 10 ppb), with (**b**) seasonal features of ΔO_3_ in aerosol enhancement at Syowa Station during 1997–2011 (see Table S1). In Fig. 3a, “t” and “p” respectively denote *t*-values and *p*-values of *t*-tests. Degrees of freedom for the *t*-test were 148,180.

Satellite BrO measurements are difficult to take at Syowa during June through early September because of the low elevation angle of sunlight. Therefore, some other proxy is needed to elucidate the relation between BrO_x_ cycles and SSA during the polar sunrise. Considering that BrO_x_ destroys surface O_3_ by R1, we attempt to identify BrO enhancement by the occurrence of low O_3_ episodes (LOEs). Here, we define LOEs as cases of O_3_ depletion amounts (ΔO_3_) larger than 10 ppb relative to the background levels, except for local contamination. Although aerosol concentrations increased with the occurrence of blowing snow (Fig. 1), ΔO_3_ in the storm conditions was 5.6 ± 2.9 ppb on average. Aerosol data during the LOEs (Fig. 3) did not include data with blowing snow. Therefore, this comparison implies that LOEs occurred considerably more often in times of high aerosol concentrations (Fig. 3a). Furthermore, major aerosol constituents in ultrafine (*D*_p_ < 0.2 μm) − fine (*D*_p_ = 0.2–2.0 μm) − coarse (*D*_p_ > 2.0 μm) modes were sea-salts that had originated from sea-ice areas, particularly in AECs^11,20^. Therefore, LOEs and BrO enhancement at Syowa might be linked closely to SSA enhancement. Actually, AECs appeared in May–December (frequently in June–October) at Syowa. During the polar night, surface O_3_ dropped only slightly in AECs (Fig. 3b). This result is likely attributable to low BrO_x_ concentrations in the atmosphere by reduction of the following photochemical reactions during times of less solar radiation, in spite of a large dispersion of SSA.

R6 $${{\rm{Br}}}_{{\rm{2}}}+{\rm{hv}}\to {\rm{2Br}}$$

R7 $${\rm{BrO}}+{{\rm{HO}}}_{{\rm{2}}}\to {\rm{HOBr}}+{{\rm{O}}}_{{\rm{2}}}$$

Data show that ΔO_3_ tended to increase from early polar sunrise (July) through August. In August, the largest ΔO_3_ reached 34 ppb, corresponding to nearly complete O_3_ depletion at Syowa. Although larger ΔO_3_ continued into September, ΔO_3_ began to decrease gradually after October when sea-ice starts melting off Syowa. Consequently, LOE, AEC, and BrO enhancement might occur simultaneously in July–October.

### Seasonal variation of constituents of blowing snow and aerosols

**Figure 4:**
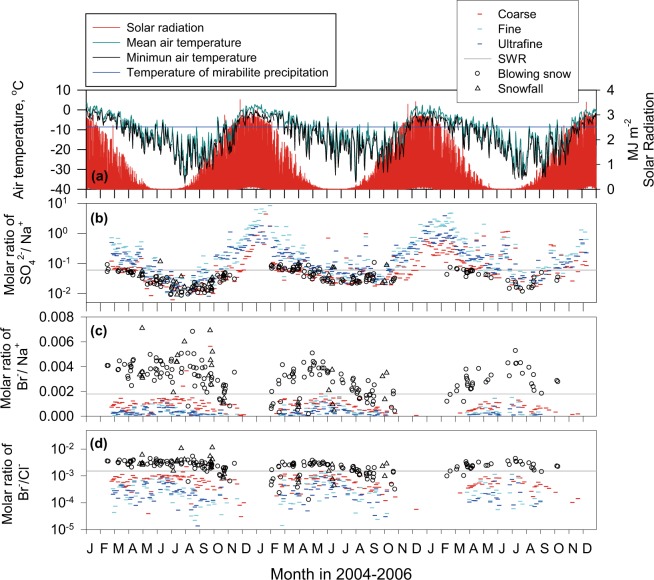
Seasonal features of air temperature, solar radiation, molar ratios of SO_4_^2−^/Na^+^, Cl^−^/Na^+^, Br^−^/Na^+^, and Mg^2+^/Na^+^ in blowing snow and aerosols at Syowa Station, Antarctica. The blue line in (**a**) shows temperatures for mirabilite precipitation. Gray lines in (**b–d**) show bulk seawater ratios.

Molar ratios of SO_4_^2−^/Na^+^ in blowing snow and aerosols were lower than SWR in April–October (Fig. 4) because of sea-salt fractionation on sea-ice^14,15^. Additionally, SO_4_^2−^/Na^+^ ratios in blowing snow matched those in aerosols well. The daily minimum of air temperature at Syowa Station dropped to temperature for mirabilite precipitation (ca. −8 °C) in mid-February and March. Although mirabilite is expected to be precipitated on new sea-ice in mid-February and March, high nss-SO_4_^2−^ concentrations might mask evidence of SO_4_^2−^ depletion in aerosols and blowing snow. Therefore, sea-salts in blowing snow and aerosols were likely to be supplied from sea-ice areas by strong winds. Br^−^/Na^+^ ratios can be changed by (1) Br^−^ enrichment by sea-salt fractionation, (2) Br^−^ loss by heterogeneous reactions, and (3) uptake/deposition of BrO_x_ generated via bromine depletion in sea-salts and oxidation of short-lived bromocarbons such as CHBr_3_. Because mirabilite (Na_2_SO_4_ 10H_2_O; below −8 °C) and hydrohalite (NaCl 2H_2_O; below −22 °C) are precipitated in/on sea-ice^15^, Br^−^/Na^+^ ratios increase gradually with sea-salt fractionation. Assuming only the occurrence of mirabilite precipitation, Br^−^/Na^+^ ratios changed from seawater ratio (0.0017) to 0.0020 (Supplementary), which was lower than the ambient ratios found for blowing snow. During our measurements, air temperature dropped to −37 °C at Syowa. When temperature near the snow and sea-ice surface reach to −25–−26 °C, the Br^−^/Na^+^ ratios can elevate to 0.004–0.005 by hydrohalite precipitation (Fig. S8). This coincidence implies strongly that sea-salt fractionation engendered Br^−^ enrichment (high Br^−^/Na^+^ ratios). Indeed, earlier studies^21,22^ have found substantial contributions of sea-salt bromines in the Antarctic troposphere. Uptake/deposition of BrO_x_ can modify Br^−^/Na^+^ ratios in the surface snow, blowing snow, and snowfall. BrO_x_ concentrations were minimal in winter and increased in spring–autumn at Dumont d’Uville Station, Antarctica^23^. However, our estimation (Supplementary) showed that ambient BrO_x_ concentrations were too low to make an important contribution to Br^−^/Na^+^ ratios in blowing snow. In addition, the relation between Na^+^ and Br^−^/Na^+^ ratios in blowing snow and snowfall shows larger variation of Br^−^/Na^+^ ratios in snowfall samples with lower Na^+^ concentrations and correlation (*R*^2^ = 0.48) in blowing snow with high Na^+^ concentrations (Supplementary). Because of the correlation and high Na^+^ concentrations in blowing snow, the impact of BrO_x_ uptake/deposition on Br^−^/Na^+^ ratios in blowing snow might be small or slight during winter–spring. Consequently, we conclude that sea-salt fractionation promotes dominant to high Br^−^/Na^+^ ratios in blowing snow.

Furthermore, Br^−^/Na^+^ ratios in both blowing snow and aerosols showed a maximum level during May–July, except in the winter of 2004, when fast sea-ice in front of Syowa broke and flowed in March and August. In contrast to strong Br^−^ enrichment in blowing snow, Br^−^/Na^+^ ratios in aerosols decreased to become lower than SWR throughout the year, particularly after early July. These seasonal features of Br^−^/Na^+^ ratios in aerosols were consistent with those at Dumont d’Uville Station, Antarctica^23^. The heterogeneous reactions (R2–5) might drive the Br^−^ release from SSA. Considering the marked Br^−^ enrichment that is found in blowing snow (Figs 2 and 4), Br^−^ might be enriched in SSA at the moment of release from the sea-ice area. Consequently, efficient Br^−^ depletion from SSA might be promoted immediately after SSA release into the atmosphere. Therefore, a large amount of BrO_x_ can be released from SSA via heterogeneous reactions.

### Estimation of BrOx released from SSA

Previous works^2,6,9^ estimated a dominant contribution of sea-ice-origin SSA and blowing snow to BrO_x_. The Br^−^ enrichment in blowing snow and strong Br^−^ depletion in SSA were not considered in those estimations. For that reason, the SSA contribution to atmospheric BrO_x_ might be underestimated. To estimate the release amounts of BrO_x_ from SSA, we assumed the following initial conditions.

1. Initial molar ratios of Br^−^/Na^−^ in SSA:
  Br^−^/Na^+^ = 0.0050 (maximum ratio of blowing snow during the winter), and
  Br^−^/Na^+^ ratios of blowing snow in each case.
2. Br^−^/Na^+^ ratios in SSA; ambient ratios and complete Br^−^ loss from SSA.
3. Molecular weight of sea-salts: 62.288 g mol^−1^, as estimated from SWR^24^.

Because of assumption^2^, the estimated value in the complete Br^−^ loss is equivalent to the upper limit of the BrO_x_ release from SSA. Data show that Na^+^ concentrations were 50–255 nmol m^−3^ (3.2–15.9 μg m^−3^ in SSA mass concentration) during AECs (Fig. S9). Aerosol particles were measured and collected in the observatory after passage through the air inlet and tubes. Therefore, the aerosol concentrations can be underestimated particularly in particles larger than 5 μm. From comparison of aerosol number concentrations measured in and outside the observatory, the underestimated mass concentrations in aerosol samples were estimated as 5–20 μg m^−3^ depending on the aerosol number concentrations (Supplementary). Therefore, we assumed SSA mass concentrations in *D* ≤ 10 μm of 7.9–46.4 μg m^−3^ in AECs (Table S2). In this range, the released BrO_x_ amounts can be estimated as 9–56 pptv (Br^−^/Na^+^ = 0.0032) and 12–70 pptv (Br^−^/Na^+^ = 0.0050) in the case of complete Br^−^ release from SSA (Fig. S12). The estimated levels were consistent with the sum concentrations of BrO_x_ (Br_2_, BrCl, and BrO) measured at Halley^10,25^. The major BrO_x_ species are Br_2_ and BrO, which can be mutually converted after photolysis and reaction with O_3_ under UV radiation. VCD_BrO_ depends on the aerosol enhanced layer thickness. The aerosol thickness of the enhanced layer was 2–4 km over Syowa Station^26^. Moreover, vertical profiles of potential temperature imply that the thickness reached 3 km on 28–29 September 2005 (Fig. S13). In the case of SSA mass concentrations (12 μg m^−3^) and the thickness (3 km) on 28 September 2005, ambient amounts of BrO_x_ can be estimated as 1.2 × 10^14^ mole cm^−2^ (Br^−^/Na^+^ = 0.0032) and 1.7 × 10^14^ mole cm^−2^ (Br^−^/Na^+^ = 0.0050). These values were well matched to the observed VCD_BrO_ (1 × 10^14^ mole cm^−2^) on 28–29 September 2004 by SCIAMACHY around Syowa. Similarly, BrO_x_ amounts in the other AECs in 2004–2006^12^ (Table S2) can be estimated as 6.9 × 10^13^–1.7 × 10^14^ mole cm^−2^ using ambient Br^−^/Na^+^ ratios of blowing snow and aerosols. These values were similar to the BrO_x_ concentrations at Halley^10,25^. Therefore, the agreement suggests that the BrO_x_ release from SSA has a greater contribution than that suggested by earlier studies^9^. Furthermore, higher Br^−^/Na^+^ ratios of blowing snow are likely to enhance activation of BrO_x_ cycles and O_3_ depletion in August at Syowa. Considering pH in blowing snow and the weaker anthropogenic impact leading to low concentrations of acidic species in the Antarctica, heterogeneous reactions on SSA after dispersion to the atmosphere were likely to be necessary processes for the atmospheric BrO_x_ cycle at the Antarctic coasts. Consequently, our results provide direct evidence that SSA dispersion from sea-ice by strong winds and the heterogeneous reactions on SSA play crucially important roles as the initial step to activate atmospheric BrO_x_ cycles at the Antarctic coasts during the polar sunrise.

Atmospheric sea-salt and halogen chemistry are important processes affecting atmospheric sea-salt and BrO_x_ cycles, which are associated closely with O_3_ and Hg depletion, and with DMS oxidation. Results show that SSA can be dispersed not only to the boundary layer but also to the free troposphere, at approx. 4 km altitude^26^. The SSA dispersion engenders modification of the atmospheric chemistry such as oxidation capacity related to O_3_ and BrO in the Antarctic free troposphere during the polar sunrise. Furthermore, multi-year sea-ice cover declines gradually with climate change (global warming), particularly in the Arctic. Actually, Br^−^ enrichment proceeds on one-year sea-ice by sea-salt fractionation^16^. Therefore, atmospheric sea-salt and BrO_x_ cycles are expected to change in polar regions through the complex linkage of ocean, sea-ice/snow, and the atmosphere.

## Methods and Analysis

### Sampling of blowing snow and snowfall

Pre-cleaned polypropylene cuboid bottles (500 ml) were used for sampling of blowing snow. The bottle was turned perpendicularly and set on a balustrade (ca. 3 m above ground/snow surface) of the atmospheric observatory, facing the wind direction in conditions of blowing snow and strong winds. Using our blowing snow sampling procedure, we were unable to segregate blowing snow particles and snowfall particles. Winds from clean air sectors accompanied the blowing snow at Syowa Station. Therefore, local contamination was not mixed in the samples. Snowfall was collected in pre-cleaned polyethylene bags fixed in a plastic container on the roof of the atmospheric observatory under calm wind conditions with snowfall and without blowing snow. Our measurements taken in 2004–2006 were of 180 and 33 samples of blowing snow and snowfall (78, 67, and 35 samples of blowing snow in 2004, 2005, and 2006; 22, 11, and 0 samples of snowfall in 2004, 2005, and 2006). Blowing snow and storm events occurred a few times a week during March–October at Syowa Station in 2004–2006. Therefore, blowing snow samples were taken in most blowing snow events at Syowa Station during our measurements. Weather conditions were usually stable from November until mid-February at Syowa Station. Therefore, blowing snow samples could not be collected in this study in November – mid-February. Both samples were melted in the observatory immediately after sampling, and were transferred to 14 ml polypropylene vials with an airtight cap. They were kept in the freezer until analysis at our laboratory in Japan. Using surplus solutions, pH and conductivity were measured using a portable pH meter (B-212; Horiba Ltd.) and a conductivity meter (B-173; Horiba Ltd.) at the Syowa Station observatory.

### Sampling of aerosols

Routine size-segregated aerosol samples were collected for 2–3 days using a mid-volume-impactor (MVI) and a back-up filter holder. Cut-off diameters were 2 and 0.2 μm. A Nuclepore filter (25 mmϕ, 110606; Whatman plc.) and PTFE filter (47 mmϕ, J100047A; Advantec MFS Inc.) were used respectively as sample substrates for MVI and back-up filtering. Along with the routine sampling, additional aerosol sampling was made daily in the aerosol enhanced conditions using the same MVI system. To avoid local contamination from the main area of Syowa Station, aerosol sampling was done only in winds from the clean air sector using a wind selector at the clean air observatory ca. 400 m distant from the main area. Aerosol samples (filters) were moved to 14 ml polypropylene vials with an airtight cap immediately after sampling. They were subsequently kept in a freezer until analysis in Japan.

### Sample analysis of blowing snow and aerosols

Samples of blowing snow and snowfall were melted at room temperature (ca. 20 °C). Because of the high concentrations of blowing snow samples, the samples were diluted 10^2^–10^3^ times using ultrapure water in reference to their conductivity. Sample solutions were filtered using disposable filters (0.45 μm pore, DISMIC-13HP; Advantec MFS Inc.) before analysis. Then, water-soluble constituents in blowing snow and snowfall were determined using ion chromatography (DX-120; Dionex Corp.). Furthermore, water-soluble aerosol constituents were determined using ion chromatography after extraction using 14 ml of ultrapure water. Analytical procedures and conditions were set as described for our previous work^12,27^.

### Measurements of aerosol number concentrations and size distributions

Aerosol number concentrations and size distributions were monitored using an optical particle counter (OPC: TD-100; Sigma Tech.) and a condensation particle counter (CPC: 3010; TSI Inc.) at Syowa Station, Antarctica from February, 1996. Measured size ranges were *D*_p_ (diameter) >0.3, >0.5, >1.0, >2.0, >3.0, and >5.0 μm in OPC. Using the CPC, we measured the aerosol number concentrations of condensation nuclei with sizes larger than 10 nm diameter. After OPC and CPC were installed at the atmospheric observatory in February, 1996–January, 2004, they were operated in the clean air observatory from February, 2004. Details of OPC specifications were presented in an earlier report^13^. Locally contaminated data were filtered by standard deviation in 10-min averages with wind data, as described for our earlier work^12^.

### Ozone measurements

Monitoring of surface O_3_ was conducted using a UV photometer (Model 1100; Tokyo Dylec Corp.) at Syowa Station, Antarctica since February, 1997 by the Japan Meteorological Agency. From February, 1997 through January, 2007, O_3_ concentrations were measured at the meteorological observatory. O_3_ measurements were made at a clean air observatory from February, 2007. Similar to aerosol data screening, locally contaminated data were filtered by standard deviation in reference to 10-min average and wind data.

### Tropospheric BrO from satellites

Tropospheric BrO vertical column densities (VCDs) from SCIAMACHY onboard ENVISAT were retrieved during 2004–2006 using the algorithm developed by the Belgian Institute for Space Aeronomy (IASB-BIRA)^28^.

## Electronic supplementary material

Supplementary Information
