# Supplementary material for: Important contributions of sea-salt aerosols to atmospheric bromine cycle in the Antarctic coasts
Source: Sci Rep. 2018 Sep 14;8:13852. doi: 10.1038/s41598-018-32287-4 (PMC6138753; doi:10.1038/s41598-018-32287-4)
Supplement: Supplementary file 1 — Supplementary Information [file 41598_2018_32287_MOESM1_ESM.docx]

**Supplementary Information**

**Important contributions of sea-salt aerosols to atmospheric bromine cycle in the Antarctic coasts**

K. Hara^1^, K. Osada^2^, M. Yabuki^3^, H. Takashima^1^, N. Theys^4^, and T. Yamanouchi^5^

1: Department of Earth System Science, Faculty of Science, Fukuoka University, Fukuoka, Japan

2: Graduate School of Environmental Studies, Nagoya University, Nagoya, Japan

3: Research Institute for Sustainable Humanosphere, Kyoto University, Uji, Kyoto

4: Belgian Institute for Space Aeronomy, Brussels, Belgium

5: National Institute of Polar research, Tokyo, Japan

Correspondence and requests for materials should be addressed to K.H. (email: harakei@fukuoka-u.ac.jp)


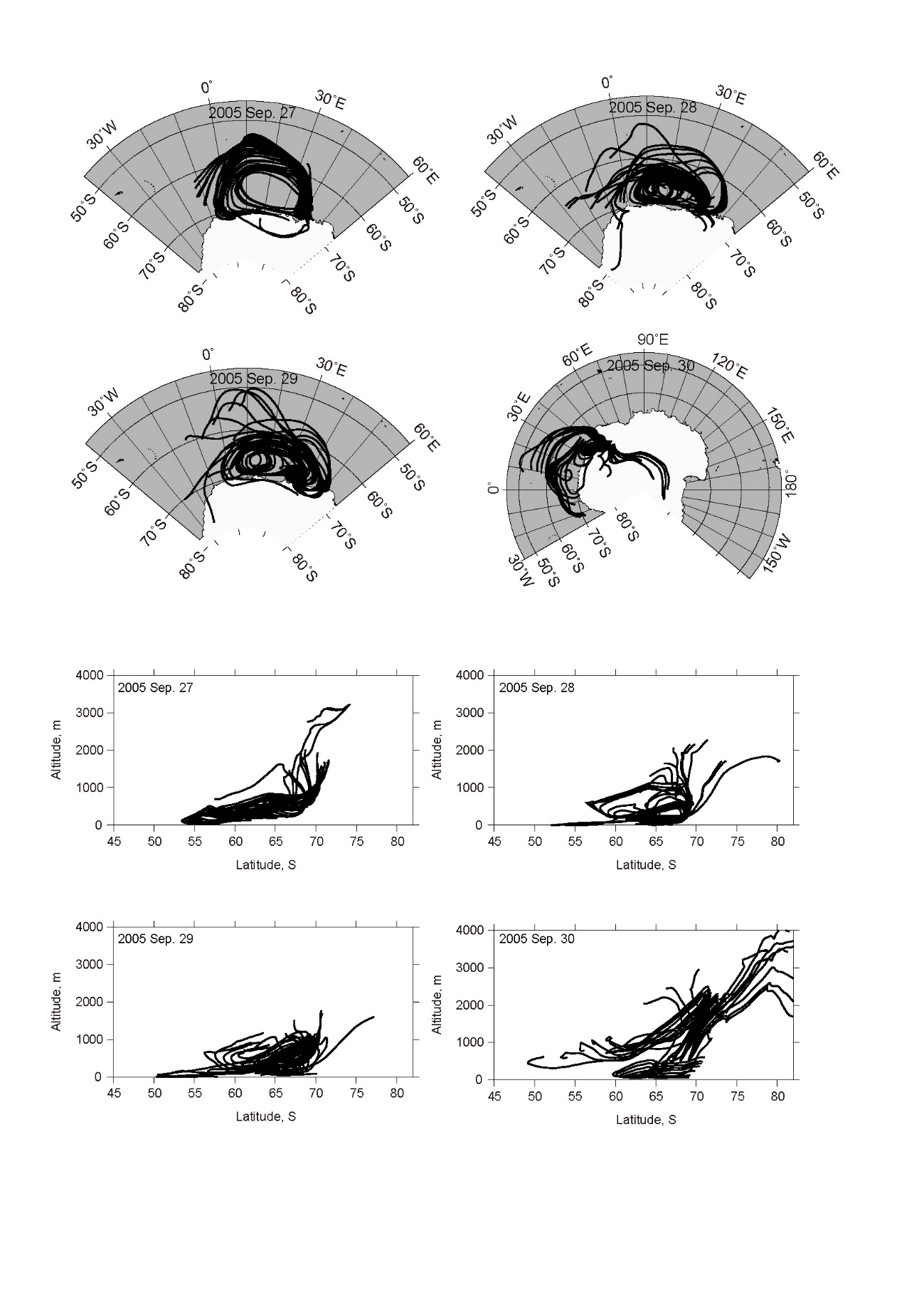


Figure S1. 5-day backward trajectory at 500 m above ground level over Lützow-Holm Bay on 27–30 September, 2005.

Figure S2. Relation between pH and conductivity in blowing snow and snowfall. In our sampling procedures, “blowing snow” samples included snowfall particles.


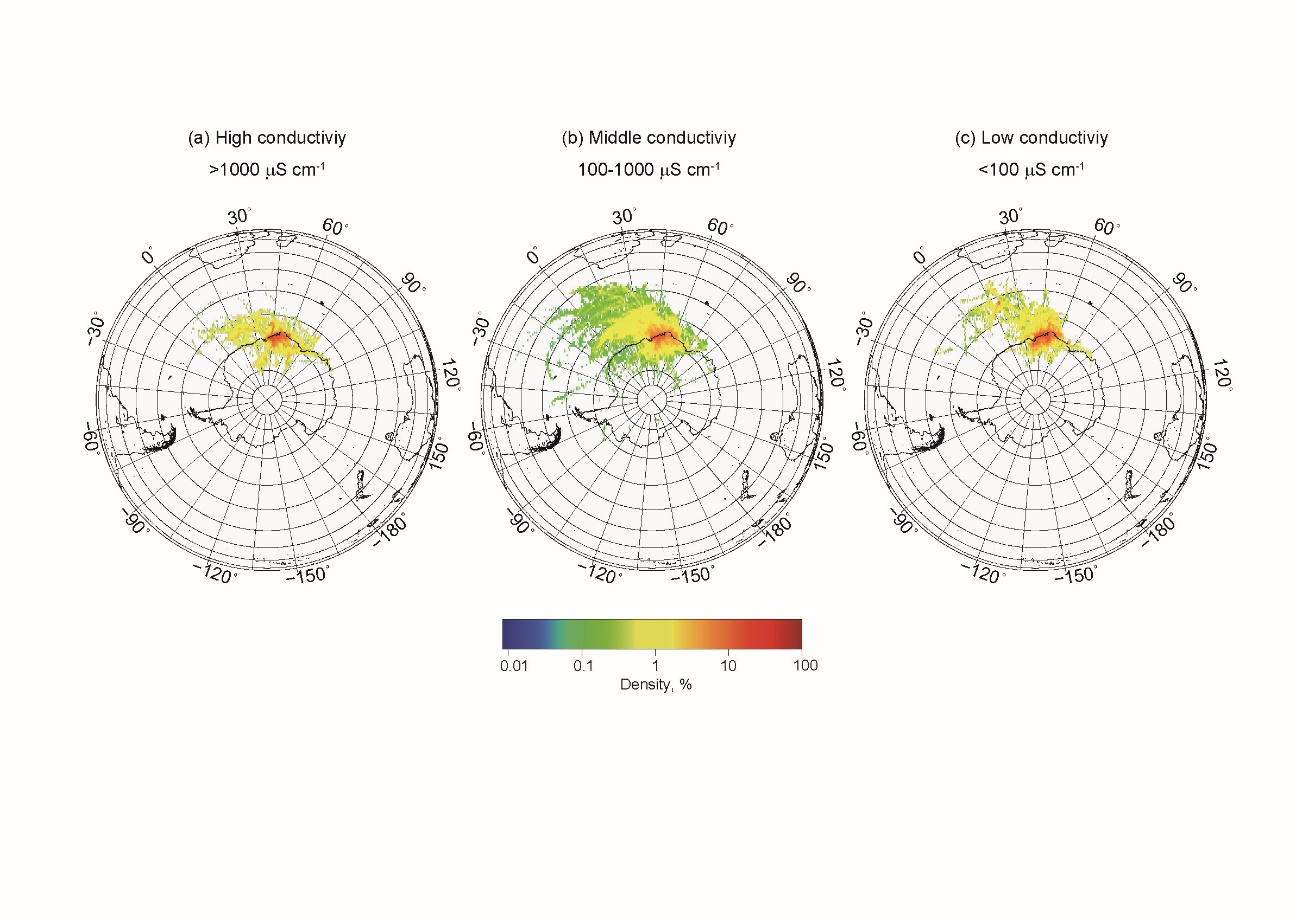
Figure S3. Distributions of air mass origins of blowing snow events with high (>1000 μS cm^-1^), middle (100-1000 μS cm^-1^), and low (<100 μS cm^-1^) conductivity. Air mass origins were computed using 5-day backward trajectories. The numbers of backward trajectories in the respective plots were 401, 1447, and 488 in high, middle, and low conductivity. It is noteworthy that we compare the density maps with density higher than 0.3% because the density maps showed different numbers of backward trajectories.

***Relation between pH and Br^-^/Na^+^ ratios in blowing snow samples***

The relation between pH and Br^-^/Na^+^ ratios in blowing snow and snowfall samples (Fig. S5) shows that Br^-^/Na^+^ ratios were lower in blowing snow samples with pH<6 than those in blowing snow samples with pH>6. Actually, the pH in blowing snow samples can be changed by (1) dilution by mixing of snowfall particles during sampling and before release from the snow surface (i.e., snowfall deposition onto snow surface on sea-ice), and (2) acidification by heterogeneous reactions on blowing snow particles and the snow surface. Figure S4 depicts Na^+^ concentrations of pH>6 and pH<6 in blowing snow samples, and of snowfall samples. This comparison indicates that Na^+^ concentrations in pH<6 of blowing snow samples decreased considerably. If acidification by heterogeneous reactions contributed substantially, then Na^+^ concentrations did not decrease markedly. Therefore, pH in blowing snow samples might result from dilution by mixing of snowfall particles. We did not segregate blowing snow particles and snowfall particles during blowing snow sampling. Therefore, ambient pH in blowing snow particles might be higher than pH in blowing snow samples. Indeed, high Br^-^/Na^+^ ratios were observed in blowing snow samples with pH>6 (Fig. S5). Furthermore, Br^-^/Na^+^ ratios in snowfall samples varied greatly compared to those of blowing snow samples with low Na^+^ concentrations and low pH (Fig. S6). Furthermore, higher Br^-^/Na^+^ ratios in blowing snow were observed in mid-February – early March when the daily minimum of air temperature was often above ca. -22 °C (hydrohalite precipitation). Therefore, high Br^-^/Na^+^ ratios in mid-February – early March might result from processes other than sea-salt fractionation, such as Br_y_ absorption on blowing snow particles and surface snow. Although Br_y_ absorption can occur in other seasons, Br_y_ concentrations were minimal in winter and were greater in spring–autumn at Dumont d’Uville Station, Antarctica^S1^. Furthermore, the relation between Na^+^ and Br^-^/Na^+^ ratios in blowing snow and snowfall shows larger variation of Br^-^/Na^+^ ratios in snowfall samples with lower Na^+^ concentrations and correlation (*R*^2^ = 0.48) in blowing snow with high Na^+^ concentrations (Fig. S7). The Na^+^ concentrations in blowing snow samples with pH>6 were 1–2 orders higher than those of snowfall samples. Because of the correlation and high Na^+^ concentrations in blowing snow, the effect of Br_y_ uptake/absorption on Br^-^/Na^+^ ratios in blowing snow might be small or slight during winter–spring.

Figure S4. Na^+^ concentrations in blowing snow samples with pH>6, blowing snow samples with pH<6, and snowfall samples, and *t*-test results. Box plots show values of 90, 75, 50 (median), 25, and 10%, represented respectively by the top bar, top box line, black middle box line, bottom box line, and bottom bar. The red lines show mean values.

Figure S5. Br^-^/Na^+^ ratios in blowing snow samples with pH>6 and pH<6, and *t*-test results. Box plots show values of 90, 75, 50 (median), 25, and 10%, represented respectively by the top bar, top box line, black middle box line, bottom box line, and bottom bar. The red lines show mean values.

Figure S6. Relation between pH and Br^-^/Na^+^ ratios in blowing snow and snowfall. The red line represents the seawater ratio (SWR).

Figure S7. Relation between Na^+^ concentrations and Br^-^/Na^+^ ratios in blowing snow and snowfall. The red line represents the seawater ratio (SWR).

***Sea-salt fractionation in blowing snow samples:***

To elucidate Br^-^ enrichment in blowing snow samples, we estimated and compared the molar ratios of Br^-^/Na^+^ in blowing snow to those reported in earlier works^S2^. We first estimated Br^-^/Na^+^ ratios using procedures from our earlier works^S3, S4^. In the case of occurrence of mirabilite precipitation only, the Br^-^/Na^+^ ratios changed from the seawater ratio (ca. 0.0017) to 0.002. Although Br^-^ can be enriched to Na^+^ concentrations by mirabilite precipitation, the estimated ratios were lower than the Br^-^/Na^+^ ratios in blowing snow samples in this study. As suggested by our earlier work^S5^, hydrohalite can be precipitated around Syowa Station, Antarctica. Then, we estimated Br^-^/Na^+^ ratios using results from laboratory experiments^S6^ and Br^-^ concentrations in seawater^S7^, as shown in Figure S8. Under the conditions with mirabilite precipitation only, Br^-^/Na^+^ ratios matched well to the value above the estimation. Below -22 °C, at which temperature hydrohalite precipitation starts, the Br^-^/Na^+^ ratios increased drastically. The values at -24 – -26 °C showed good agreement with the ratios in blowing snow samples. Although SO_4_^2-^/Na^+^ ratios in laboratory experiments^S6^ dropped to lower than 10^-2^ by hydrohalite precipitation below 250 K as shown in Fig. S8, SO_4_^2-^/Na^+^ ratios in blowing snow and aerosols ranged in the order of 10^-2^ during the winter–spring. These ratios in the order of 10^-2^ were coincident with the ratios of frost flowers, brines, and snow on seasonal sea-ice observed in Antarctic coast^S8^ and northwestern Greenland during the winter^S9^. Furthermore, sulfate particles (i.e. particles containing non-sea-salt SO_4_^2-^) were present in the atmosphere at Syowa Station even in the winter–spring^S10, S11^. Consequently, ambient SO_4_^2-^/Na^+^ ratios of blowing snow and aerosols did not dropped likely to the ratios lower than 10^-2^ in spite of occurrence of hydrohalite precipitation. Therefore, higher Br^-^/Na^+^ ratios might result predominantly from sea-salt fractionation by mirabilite and hydrohalite precipitation.

Figure S8. Temperature dependence of sea-salt concentrations and the molar ratio of Br^-^/Na^+^ in sea-salt fractionation on sea-ice. The red line represents the seawater ratio.

***Contribution of BrO_x_ deposition to Br^-^/Na^+^ ratios in blowing snow***

Gaseous BrO_x_ can be deposited onto surface snow. To elucidate the contribution of BrO_x_ deposition, we attempted to estimate the dry deposition flux (*F*_deposition_). Dry deposition flux was calculated as

$$F_{depostion}=V_{{BrO}_{x}}C_{{BrO}_{x}}$$

where *V*_BrOx_ and C_BrOx_ respectively represent the dry deposition velocity and concentrations of gaseous BrO_x_. The dry deposition velocity was assumed as 0.1 cm s-^1^, 0.5 cm s-^1^, and 1.0 cm s-^1^ based on inferences from earlier work^S1^. HBr, BrONO_2_, and HOBr were the most important BrO_x_ species in BrO_x_ dry deposition^S1, S12^. Results in a earlier report of the relevant literature^S12^ suggest dry deposition velocity of 0.96 m s-^1^ for HBr and BrONO_2_ and 0.4 m s-^1^ for HBr. Consequently, the estimated values were likely the upper limit of dry deposition flux. The range of BrO_x_ concentrations was 0.01–1.0 nmol m^-3^, considering our observations and the range observed at Dumont d’Urville^S1^. Furthermore, model estimation expected that dominant BrO_x_ species were Br_2_ in winter, and HBr and HOBr in spring^S1^. In the case of C_BrOx_ = 0.1 nmol m^-3^ and *V*_Bry_ = 1.0 cm s-^1^, *F*_deposition_ was 8.6–86.4 nmol m^-2^ day^-1^. This value corresponds to the deposition flux under conditions with typically high BrO_x_ concentrations and high deposition. When BrO_x_ is deposited onto the snow surface with Na^+^ concentrations of 10–10^4^ μmol L^-1^, the Br^-^/Na^+^ ratios in the surface snow of 1 m (width) × 1 m (width) × 1 cm (depth) can change in scale of 8.6 × 10^-4^ – 8.6 × 10^-7^ day^-1^. This estimation suggests that dry deposition of BrO_x_ has an important contribution only to low salinity snow such as snowfall with Na^+^ concentration of ≤10 μmol L^-1^. Considering that Na^+^ concentrations were 10^3^–10^4^ μmol L^-1^ in blowing snow, the effect of BrO_x_ dry deposition might be too low to modify Br^-^/Na^+^ ratios of blowing snow. Therefore, high Br^-^/Na^+^ ratios in blowing snow during winter – spring might result from Br^-^ enrichment by sea-salt fractionation. During the autumn of 2005 and 2006, Br^-^/Na^+^ ratios were similar to the SWR, whereas Br^-^/Na^+^ in 2004 autumn were remarkably high compared to SWR. Such high Br^-^/Na^+^ ratios cannot be explained by sea-salt fractionation attributable to higher air temperature for precipitation of mirabilite and hydrohalite. Therefore, high Br^-^/Na^+^ ratios in 2004 autumn might result from processes other than sea-salt fractionation, perhaps from BrO_x_ absorption on blowing snow particles and surface snow. As described above, however, uptake/deposition of BrO_x_ has less contribution also in 2004 autumn because of the lower BrO_x_ concentration in the autumn than in spring–summer^S1^. In contrast to autumns in 2005 and 2006, the open sea surface appeared on the windward side of Syowa Station in March, 2004 when sea-ice was broken and flowed out by strong winds in the storms. Then, new sea-ice was formed again immediately after weather recovery. Consequently, the drastic change of sea-ice conditions might be related to the high Br^-^/Na^+^ ratios in blowing snow, although specific processes for the high Br^-^/Na^+^ ratios were unclear.

***Correction of mass concentrations of sea-salt aerosols:***

We must consider the particle loss by aerosol inlet system in measurements of aerosol number concentrations because aerosol particles can be trapped onto the wall by impaction and segregation in the inlet depending on the wind speed. To estimate ambient aerosol number concentrations, aerosol-passing efficiency to OPC was estimated using simultaneous measurements with balloon-borne OPC, portable OPC, and OPC at the observatory^S13, S14, S15^. The aerosol number concentrations were measured under dry conditions (low relative humidity) because OPCs were operated in the observatory with air conditioning and in insulator box with small heater outside the observatory to keep a suitable operating temperature. Because of the low number concentrations in coarse mode, aerosol counts were cumulative over one hour. Then, the aerosol number concentrations were estimated. The aerosol-passing efficiencies in our measurement condition were 94% in *D*_p_ > 0.3 μm, 93% in *D*_p_ > 0.5 μm, 88% in *D*_p_ > 1.0 μm, 87% in *D*_p_ > 2.0 μm, and 1% in *D*_p_ > 5.0 μm. Considering the cumulated aerosol counts in *D*_p_ > 5.0 μm greater than 500 in the ambient air during simultaneous measurements, uncertainty might be less than 10%. Then, ambient aerosol number concentrations were calculated from the number concentrations measured using the OPC at the clean air observatory (as a part of aerosol monitoring measurements at Syowa Station). Ambient aerosol mass concentrations were calculated based on the assumption that aerosol particles were spherical, with density of 2.0 g cm^-3^ (corresponding to NaCl). Indeed, individual aerosol particle analysis indicated that sea-salt particles were deliquescent, i.e. spherical^S11^, although the aerosol number concentrations were measured under dry conditions. Furthermore, spherical shape has the smallest volume on particle size compared to other shapes such as cuboid. Therefore, the assumption might be reasonable and proper. The underestimated mass depends strongly on the aerosol number concentrations and size distributions in AECs, as shown in Figure S10. The underestimated mass concentrations were distributed dominantly in coarse mode, particularly in aerosols with *D*_p_ > 5.0 μm (Fig. S11). Therefore, aerosol mass concentrations were corrected in each case of the AECs based on the aerosol number concentrations and size distributions (Table S2).

Figure S9. Seasonal features of Na^+^ concentrations in aerosols at Syowa Station, Antarctica during February 2004 – December 2006. Resolution of routine aerosol sampling was 2–3 days depending on weather conditions.

Figure S10. Relation between the corrected aerosol number concentrations and the underestimated aerosol mass concentrations in AECs.

Figure S11. Contribution of the underestimated mass concentrations of aerosols in AECs.

Figure S12. Relation between SSA mass concentrations and the upper limit of BrO_x_ concentrations.

Figure S13. Vertical profiles of potential temperature over Syowa Station, Antarctica on 28–29 September, 2005. Arrows indicate the top height of the aerosol-enhanced layer.

Table S1. Aerosol enhanced episodes* at Syowa Station, Antarctica

| Start time (UT) | End time (UT) | Duration (hr) |
| --- | --- | --- |
| 1997/8/3 23:38 | 1997/8/5 4:22 | 28.7 |
| 1997/8/28 4:35 | 1997/8/29 3:44 | 23.2 |
| 1997/10/24 13:13 | 1997/10/25 21:50 | 32.6 |
| 1998/7/29 0:15 | 1998/8/1 18:20 | 90.1 |
| 1998/8/30 8:02 | 1998/9/3 23:07 | 111.1 |
| 1998/9/15 17:02 | 1998/9/18 5:07 | 60.1 |
| 1998/10/1 23:02 | 1998/10/3 17:02 | 42.0 |
| 1998/10/10 5:02 | 1998/10/11 17:02 | 36.0 |
| 1999/5/13 4:20 | 1999/5/13 15:58 | 11.6 |
| 1999/5/14 13:05 | 1999/5/16 2:26 | 37.4 |
| 1999/7/23 15:36 | 1999/7/24 12:43 | 21.1 |
| 1999/8/15 3:04 | 1999/8/16 6:33 | 27.5 |
| 2000/8/8 8:13 | 2000/8/9 16:46 | 32.5 |
| 2000/10/10 3:27 | 2000/10/10 17:30 | 14.0 |
| 2000/10/16 21:32 | 2000/10/18 0:30 | 27.0 |
| 2000/10/31 12:36 | 2000/11/1 9:22 | 20.8 |
| 2001/10/3 9:00 | 2001/10/4 23:30 | 38.5 |
| 2001/10/20 15:03 | 2001/10/25 7:10 | 112.1 |
| 2001/10/28 6:36 | 2001/10/28 18:20 | 11.7 |
| 2001/12/16 0:52 | 2001/12/18 22:14 | 69.4 |
| 2002/7/19 17:00 | 2002/7/20 0:00 | 7.0 |
| 2002/7/29 14:20 | 2002/7/30 10:30 | 20.2 |
| 2002/8/7 19:00 | 2002/8/8 7:30 | 12.5 |
| 2002/8/18 17:20 | 2002/8/19 18:10 | 24.8 |
| 2002/8/27 5:28 | 2002/8/27 16:05 | 10.6 |
| 2002/8/29 6:00 | 2002/8/30 7:46 | 25.8 |
| 2002/9/3 5:03 | 2002/9/4 6:08 | 25.1 |
| 2002/10/7 10:00 | 2002/10/10 14:16 | 76.3 |
| 2003/5/3 18:30 | 2003/5/4 9:45 | 15.2 |
| 2003/7/5 22:30 | 2003/7/7 0:50 | 26.3 |
| 2003/8/8 4:00 | 2003/8/9 14:00 | 34.0 |
| 2003/8/9 22:25 | 2003/8/10 10:54 | 12.5 |
| 2003/8/12 3:25 | 2003/8/13 7:15 | 27.8 |
| 2003/9/16 15:00 | 2003/9/17 12:00 | 21.0 |
| 2003/11/21 23:00 | 2003/11/22 9:20 | 10.3 |
| 2004/6/17 10:50 | 2004/6/18 20:00 | 33.2 |
| 2004/7/10 13:00 | 2004/7/12 4:30 | 39.5 |
| 2004/7/27 10:38 | 2004/7/29 1:30 | 38.9 |
| 2004/8/7 4:30 | 2004/8/8 4:50 | 24.3 |
| 2004/8/19 19:48 | 2004/8/20 18:17 | 22.5 |
| 2004/9/26 1:43 | 2004/9/26 15:20 | 13.6 |
| 2005/5/24 23:00 | 2005/5/27 23:50 | 72.8 |
| 2005/7/20 0:00 | 2005/7/21 10:00 | 34.0 |
| 2005/8/9 16:00 | 2005/8/11 0:30 | 32.5 |
| 2005/9/27 0:00 | 2005/9/28 18:05 | 42.1 |
| 2006/8/9 10:30 | 2006/8/10 5:00 | 18.5 |
| 2006/9/4 8:30 | 2006/9/5 7:30 | 23.0 |
| 2007/9/8 15:14 | 2007/9/10 17:44 | 50.5 |
| 2007/10/16 11:05 | 2007/10/16 18:15 | 7.2 |
| 2008/6/27 2:54 | 2008/6/27 15:35 | 12.7 |
| 2008/7/6 3:54 | 2008/7/7 4:04 | 24.2 |
| 2009/6/7 2:44 | 2009/6/7 22:23 | 19.6 |
| 2009/6/9 12:43 | 2009/6/11 7:15 | 42.5 |
| 2009/7/18 10:35 | 2009/7/19 18:45 | 32.2 |
| 2009/7/19 23:54 | 2009/7/21 0:44 | 24.8 |
| 2009/7/21 14:35 | 2009/7/22 2:15 | 11.7 |
| 2009/8/18 16:45 | 2009/8/20 19:24 | 50.7 |
| 2009/9/12 8:55 | 2009/9/14 6:44 | 45.8 |
| 2009/10/26 3:34 | 2009/10/26 20:24 | 16.8 |
| 2010/6/22 14:44 | 2010/6/24 9:25 | 42.7 |
| 2010/7/7 18:24 | 2010/7/8 17:25 | 23.0 |
| 2010/8/20 7:05 | 2010/8/22 1:05 | 42.0 |
| 2010/8/23 5:54 | 2010/8/23 21:45 | 15.8 |
| 2010/9/5 13:54 | 2010/9/7 10:05 | 44.2 |

*: Aerosol enhancement conditions were defined as conditions with the absence of blowing snow and higher aerosol number concentrations of *D*_p_ > 0.3 μm than 10 particle cm^-3^, corresponding to approximately 90 percentile values of the aerosol number concentrations at Syowa Station.

Table S2. Estimated BrO_x_ in our dataset with both blowing-snow samples and additional aerosol samples in 2004–2006

| Date | Blowing snow | Aerosols | | | Corrected SSA mass concentration* | Aerosol layer height | Ambient** | Potential*** | ΔO_3_ |
| --- | --- | --- | --- | --- | --- | --- | --- | --- | --- |
|  |  | *D*>2 μm | *D*: 0.2–2 μm | *D*<0.2 μm |  |  | VCD_BrOx_ | VCD_BrOx_ |  |
|  | Br^-^/Na^+^ | Br^-^/Na^+^ | Br^-^/Na^+^ | Br^-^/Na^+^ | μg m^-3^ | m | mole cm^-2^ | mole cm^-2^ | ppb |
| 2004 Aug. 20 | 0.0050 | 0 | 0 | 0 | 7.9–14.4 | 2000 | 7.6 × 10^13^ –  1.4 × 10^14^ | 7.6 × 10^13^ –  1.4 × 10^14^ | 5.7 |
| 2004 Sep. 26 | 0.0041 | 0 | 0 | 0 | 11.1–26.5 | 2000 | 8.8 × 10^13^ –  2.1 × 10^14^ | 8.8 × 10^13^ –  2.1 × 10^14^ | 13.4 |
| 2005 Aug. 10 | 0.0032 | 0.00052 | 0.00003 | 0 | 16.0–46.4 | 2500 | 1.1 × 10^14^ –  3.5 × 10^14^ | 1.2 × 10^14^ –  3.6 × 10^14^ | 7.0 |
| 2005 Sep. 28 | 0.0040 | 0.00015 | 0 | 0 | 10.4–35.2 | 3000 | 1.2 × 10^14^ –  3.2 × 10^14^ | 1.2 × 10^14^ –  3.3 × 10^14^ | 16.0 |
| 2006 Aug. 9 | 0.0039 | 0 | 0 | 0 | 8.5–16.5 | 2200 | 7.1 × 10^13^ –  1.4 × 10^14^ | 7.1 × 10^13^ –  1.4 × 10^14^ | 11.0 |

*: Corrected mass concentrations were estimated from each aerosol size distribution measured by OPC.

**: VCD_BrOx_ was estimated using ambient molar ratios of Br^-^/Na^+^ in aerosols.

***: Potential VCD_BrOx_ was estimated in the case of complete Br^-^ depletion from aerosols.

**References**

S1. Legrand, M., Yang, X., Preunkert, S. & Theys, N. Year-round records of sea salt, gaseous, and particulate inorganic bromine in the atmospheric boundary layer at coastal (Dumont d’Urville) and central (Concordia) East Antarctic sites. *J. Geophys. Res.* **121**, 997-1023 (2016).

S2. Koop, T., Kapilashrami, A., Molina, L. & Molina, M. Phase transitions of sea-salt/water mixtures at low temperatures: Implications for ozone chemistry in the polar marine boundary layer. *J. Geophys. Res.* **105**, 26393-26402 (2000).

S3. Hara, K. et al. Haze episodes at Syowa Station, coastal Antarctica: Where did they come from? *J. Geophys. Res.* **115**, D14205, doi:10.1029/2009JD012582 (2010).

S4. Hara, K.. Matoba, S., Hirabayashi, M. & Yamasaki, T. Frost flowers and sea-salt aerosols over seasonal sea-ice areas in north-western Greenland during winter–spring. *Atmos. Chem. Phys.* **17**, 8577-8598 (2017).

S5. Hara, K. Osada, K., Yabuki, M. & Yamanouchi, T. Seasonal variation of fractionated sea-salt particles on the Antarctic coast. *Geophys. Res. Lett.* **39**, L18801, doi:10.1029/2012GL052761 (2012).

S6. Richardson, C. Phase relationships in sea ice as a function of temperature. *J. Glaciol.* **17**, 507-519 (1976).

S7. Millero, F., Feistel, R., Wright, D. & McDougall, T. The composition of Standard Seawater and the definition of the Reference-Composition Salinity Scale. *Deep Sea Res. Part I: Oceanographic Research Papers* **55**, 50-72 (2008).

S8. Rankin, A., Auld, V. & Wolff, E. Frost flowers as a source of fractionated sea salt aerosol in the polar regions. *Geophys. Res. Lett.* **27**, doi:10.1029/2000GL011771 (2000).

S9. Hara, K., Matoba, S., Hirabayashi, M. & Yamasaki, T. Frost flowers and sea-salt aerosols over seasonal sea-ice areas in northwestern Greenland during winter–spring. *Atmos. Chem. Phys.* **17**, 8577–8598 (2017).

S10. Hara, K., Osada, K., Yabuki, M. & Yamanouchi, T. Seasonal variation of fractionated sea-salt particles on the Antarctic coast. *Geophys. Res. Lett.* **39**, L18801, doi:10.1029/2012GL052761.(2012).

S11. Hara, K., Osada, K. & Yamanouchi, T. Tethered balloon-borne aerosol measurements: seasonal and vertical variations of aerosol constituents over Syowa Station, Antarctica. *Atmos. Chem. Phys.* **13**, 9119-9139 (2013).

S12. Parrella, J.P. et al., V. Tropospheric bromine chemistry: implications for present and pre-industrial ozone and mercury. *Atmos. Chem. Phys.* **12**, 6723–6740 (2012).

S13. Osada, K. et al. Atmospheric observation room for clean air at Syowa Station, East Antarctica. *Antarctic Record*, **50**, 86-102, (2006) (in Japanese with English abstract)

S14. Hara, K., Osada, K., Nishita-Hara, C. & Yamanouchi, T. Seasonal variations and vertical features of aerosol particles in the Antarctic troposphere. *Atmos. Chem. Phys.* **11**, 5471-5484 (2011).

S15. Hara, K., Hayashi, M., Yabuki, M., Shiobara, M. & Nishita-Hara, C. Simultaneous aerosol measurements of unusual aerosol enhancement in troposphere over Syowa Station, Antarctica. *Atmos. Chem. Phys.* **14**, 4169-4183, doi:10.5194/acp-14-4169-2014, (2014).
